# Non Epitheliotropic B-Cell Lymphoma with Plasmablastic Differentiation vs. Cutaneous Plasmacytosis in a 12-Years-Old Beagle: Case Presentation and Clinical Review

**DOI:** 10.3390/vetsci8120317

**Published:** 2021-12-09

**Authors:** Maria Teresa Antognoni, Ambra Lisa Misia, Chiara Brachelente, Luca Mechelli, Andrea Paolini, Arianna Miglio

**Affiliations:** 1 Department of Veterinary Medicine, University of Perugia, Via San Costanzo 4, 06124 Perugia, Italy; maria.antognoni@unipg.it (M.T.A.); chiara.brachelente@unipg.it (C.B.); luca.mechelli@unipg.it (L.M.); 2 Faculty of Veterinary Medicine, University of Teramo, Via Renato Balzarini 1, 64100 Teramo, Italy; apaolini@unite.it

**Keywords:** non epitheliotropic B-cell lymphoma, cutaneous plasmacytosis, dog, flow cytometry, immunohistochemistry

## Abstract

Cutaneous lymphoid neoplasms and cutaneous plasmacytosis are rare in the dog; in human and in veterinary medicine, these have many clinical, cytological, histological, and phenotypic similarities, and a diagnosis of certainty is not easy. The aim of this study is to describe a case of cutaneous non epitheliotropic B-cell lymphoma (CNEBL) with plasmablastic differentiation vs. multiple cutaneous plasmacytosis (CP) in a dog, since the scarce bibliographic data on these topics. A 12-year-old male Beagle dog was presented for multiple, nodular, cutaneous, and subcutaneous, indolent masses disseminated on the whole body. Cytological, histological, flow cytometric, and immunohistochemical examinations, as well as complete radiographic evaluation, echocardiography, and abdominal ultrasound were performed. Cytology, histopathology, flow cytometric, and immunohistochemical examination, performed on the skin lesions, revealed a B-cell phenotype with plasmablastic differentiation. Nevertheless, a final diagnosis could not be achieved and it was categorized as a case of borderline CNEBL with plasmablastic differentiation versus CP. The dog was treated with a COP chemotherapeutic protocol. Total remission was obtained and relapse occurred 120 days later. To our knowledge, specific markers are actually unavailable to certainly differentiate CNEBL and CP in the dog and future studies are needed to improve knowledge on these pathologies in veterinary medicine, since prognosis and therapy are different.

## 1. Introduction

Canine cutaneous round cell tumors are a heterogeneous group of neoplastic diseases with different histologic origins, prognoses, and treatments. They include canine cutaneous histiocytoma, cutaneous lymphoma, plasmacytoma, and poorly differentiated mast cell tumors [1,2]. Some authors include also amelanotic melanoma, neuroendocrine tumor, transmissible venereal tumor, and histiocytic sarcoma in the differential diagnosis [1,2,3]. In many cases, due to the similar morphology of round tumor cells, the cytological and histopathological examinations are not able to obtain a definitive diagnosis, and more specific investigations are needed such as flow cytometry and immunohistochemistry [2].

Cutaneous lymphoma in dogs represents only 1% of canine skin tumors [4,5]; it can be solitary, generalized, and multifocal and it is classified in epitheliotropic and non-epitheliotropic forms based on the histological assessment of the skin lesions and the location of the neoplastic lymphocytes. Epitheliotropic cutaneous lymphoma is typical of T-cell origin and it is also known as cutaneous epitheliotropic T-cell lymphoma; it is diagnosed when neoplastic lymphocytes infiltrate the epidermis [6]. Neoplastic cells can also demonstrate tropism for hair follicles and apocrine sweat glands [7]. This disease is a rare neoplastic condition in dogs with a poor prognosis [4,6,8]. The clinical presentation is highly variable, ranging from erythema, plaques, ulcers to multiple nodules of variable size [4,6,7,8,9]. The classic neoplastic cells in canine cutaneous epitheliotropic T-cell lymphoma have a phenotype CD3^+^ (a common marker of all T lymphocytes) and, in 80% of cases, CD4^−^/CD8^+^ cytotoxic T cells [4,7,8,10]. In non-epitheliotropic cutaneous lymphoma, neoplastic lymphocytes are found mainly in the dermis and/or subcutis and the immunophenotype can be of B-cell or T-cell origin. [6] Non-epitheliotropic lymphomas can appear with dermal or subcutaneous nodules or plaques generally non pruritic, ulcerated, or alopecic with crusts. The face, lips, lower extremities, neck, and trunk are often affected [11]. Most of the canine non-epitheliotropic cutaneous lymphomas are of T-cell origin [11,12]. Non-epitheliotropic B-cell lymphomas are extremely rare both in humans and in dogs [11,12,13,14]. In veterinary literature only two case reports of cutaneous and subcutaneous non-epitheliotropic B-cell lymphoma in dogs [12,14,15] and one case in a cat [16] have been described. 

Another group of cutaneous round cell neoplasm is plasma cell tumors. Particularly, extramedullary plasmacytoma can be both cutaneous and non-cutaneous. An uncommon form of multiple cutaneous plasmacytoma that appears as red-brown plaques or raised cutaneous lesions, has been rarely described in humans [17] and in dogs [18,19] and is known as cutaneous plasmacytosis (CP). This form is characterized by the presence of multiple skin nodules of variable size, with or without systemic involvement [18,20,21]. 

Generally, it is possible to distinguish the type of round cell tumor by using cytology, histopathology, flow cytometry, and immunohistochemistry. Nevertheless, it is known to be difficult to distinguish cutaneous non epitheliotropic B-cell lymphoma (CNEBL) from multiple cutaneous plasmacytosis (CP), because of their similarity and the need to identify specific diagnostic markers.

In this report, we describe a case of canine B-cell lymphoma with plasmablastic differentiation with purely cutaneous presentation vs. CP, diagnosed at the Department of Veterinary Medicine of the University of Perugia, with the aim to improve knowledge on these very uncommon neoplastic pathologies [12,15,18,19]. 

## 2. Case Report

A 12-year-old non-neutered male Beagle was presented to the oncology service at the University of Veterinary Medicine of Perugia, Italy, for a suspected cutaneous round cell tumor. Previously, the referring veterinarian treated the dog with a non-steroid anti-inflammatory drug (meloxicam, unknown dosage) for back pain. After an initial improvement, the owner noted the presence of multiple cutaneous and subcutaneous nodules and decided for a specialist medical examination. 

**Figure 1:**
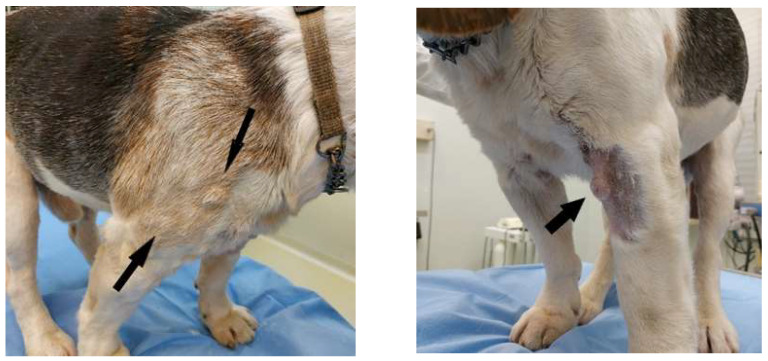
Clinical presentation of a dog with cutaneous non epitheliotropic B-cell lymphoma (CNEBL) with plasmablastic differentiation vs. multiple cutaneous plasmacytosis (CP). Note the presence of skin nodules of various sizes disseminating throughout the body (arrows).

On physical examination, the dog presented poor coat, mild dehydration, body condition score (BCS) 2, and pain on the posterior train with reduced mobility. The most important and striking clinical sign was the presence of multiple, nodular, cutaneous, and subcutaneous, indolent masses, not ulcerated and not alopecic, disseminated on the whole body, especially on forelimbs, dorsum, thorax, head, and oral mucosae (Figure 1). The diameter of lesions ranged from 5 to 12 mm. Some nodules were isolated, others formed plaques.

Clinical evaluation, laboratory analysis (Complete blood count-CBC, serum bio-chemistry, serum protein electrophoresis, antibody titers for *Leishmania* spp., *Ehrlichia* spp., *Anaplasma* spp., *Rickettsia* spp.), abdominal ultrasound, echocardiography, complete bones radiographic examination, as well as cytological, histological, and immunophenotypical analysis (flow cytometry and immunohystochemistry) of some skin lesions were performed. Results of CBC, serum chemistry panel, and serum protein electrophoresis were unremarkable except for a mild normochromic normocytic anemia (RBC:4.70 × 10^6^ μL, reference interval [RI]: 5.5–8.5 × 10^6^ μL; Sysmex XT 1800VET hematology analyzer, Sysmex, Kobe, Japan). Serology tests were negative. The abdominal ultrasound revealed that the lesions appeared as anechoic cutaneous nodules and no internal organs lesions were identified. Echocardiography examination revealed the presence of a double jet of mitral regurgitation, signs of systemic arterial hypertension, and mild pulmonary arterial hypertension.

Bones radiographic examination did not reveal osteolytic areas.

## 3. Cytology

**Figure 2:**
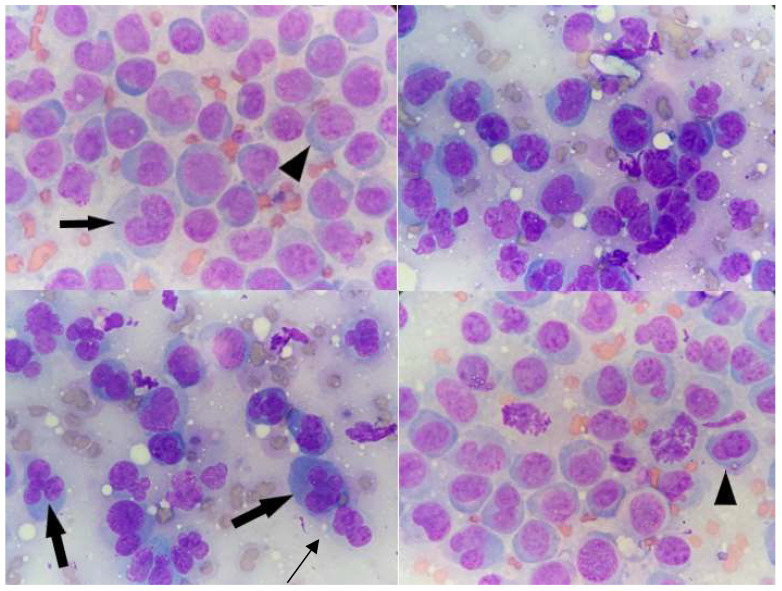
Fine needle aspiration cytology of skin nodules in a 12-year-old non-neutered male Beagle with cutaneous non epitheliotropic B-cell lymphoma (CNEBL) with plasmablastic differentiation versus multiple cutaneous plasmacytosis (CP). Note the evidence of monomorphic round cell populations of medium-large cells with multi-lobed flower-like nuclei (black arrows), reticular chromatin, and occasionally evident multiple nucleoli (arrowheads). These cells had scant to moderate basophilic to clear cytoplasm containing occasional punctate clear vacuoles (Diff-Quick, 100×).

Fine needle aspiration cytology was performed from different cutaneous lesions and the different samples showed similar findings. All smears were highly cellular with mild blood contamination and were characterized by a monomorphic cell population consisting of round elements, of variable size, with a large, round, central to eccentric, indented, occasionally kidney-shaped and sometimes multi-lobed, flower-like nucleus, with reticular chromatin, and multiple nucleoli. Cells showed a variable amount of hyper basophilic to clear cytoplasm, sometimes with lighter perinuclear area and punctate clear vacuoles. Marked anisocariosis and anisocytosis were present with occasional mitoses (1–2) at 100× magnification (Figure 2).

Cytologic evaluation suggested a round cell tumor with lymphoid, plasma cell or histiocytic origin in the differential diagnosis. Due to the non-conclusive results of cytopathological examination, histopathological and immunohistochemical investigations on skin biopsies, other than flow cytometric analysis of fine needle aspirates were performed.

## 4. Histopathology

**Figure 3:**
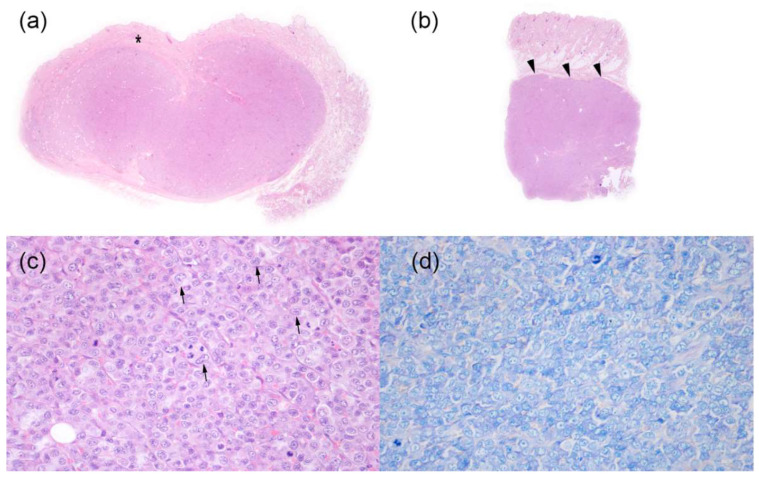
Histopathological findings of skin nodules in a 12-year-old non-neutered male Beagle with cutaneous non epitheliotropic B-cell lymphoma (CNEBL) vs. multiple cutaneous plasmacytosis (CP). (**a**) * Subgross images of one nodule from the right inguinal region. Neoplastic cells are expanding the mid and deep dermis (asterisk) extending to the subcutaneous tissue (Hematoxilin-eosin, 1.25×). (**b**) Subgross images of the nodule of the proximal region of the left paw. Neoplastic cells are invading and expanding the subcutaneous tissue (arrowheads) (Hematoxilin-eosin, 1.25×). (**c**) The tumor was composed of densely packed round cells, with eosinophilic cytoplasm, and large, frequently indented nuclei (arrows) with vesicular chromatin and prominent nucleoli (Hematoxilin-eosin, 40×). (**d**) Tumor cells do not show the presence of intracytoplasmic metachromatic granules (Giemsa, 40×).

Skin biopsies from a subcutaneous mobile nodule of the right inguinal region (1 × 1 × 0.5 cm^3^) (Figure 3a), a non-mobile nodule of the rump region (0.7 × 0.7 × 0.4 cm^3^) and a non-mobile nodule of the proximal region of the left paw (0.8 cm diameter punch) (Figure 3b) were examined. All the samples examined showed the presence of a densely cellular, poorly demarcated, non-encapsulated and infiltrative growing tumor. The tumor expanded, in one of the nodules, the thickness of the middle and deep dermis, while in the other two nodules, it extensively involved the adipose panniculus subcutaneous. The neoplasm consisted of densely packed parallel sheets of round cells with mostly distinct cytoplasmic borders, moderate amounts of weakly eosinophilic or pale and occasionally vacuolized cytoplasm, large, round, or frequently indented, central nuclei (3–4 red blood cells in size) with vesicular chromatin and sometimes prominent and multiple nucleoli (Figure 3c). Moderate anisocytosis and anisokaryosis were visible, and mitotic count was 35 mitosis/10 HPF (2.37 mm^2^). Single cell necrosis and small disseminated necrotic foci were observed in association with small hemorrhages. Epitheliotropism of neoplastic cells into the epidermis or adnexal structures was not evident. Giemsa staining of neoplastic cells was negative (Figure 3d). Based on histological examination, a diagnosis of B-cell lymphoma vs. cutaneous plasmacitosis was obtained.

## 5. Flow Cytometry

Flow cytometry of cells obtained from fine needle aspirates of nodules was performed and the antibodies panel used is summarized in Table 1. This examen revealed as follows: skin aspirate consisted mostly (74%) of large elements resulting cluster of differentiation—CD45+, CD44+, CD18+, dim CD11b+, cyCD79b+, IgG+, IgM+, IgE+, and negative for CD3, CD4, CD5, CD8 e CD21, CD14, CD34, MHCII, CD11d, and CD117. It also identified a population (9%) of very large elements CD45+ CD44+ CD18+ dim/neg cyCD79b+ and negative for all other markers used. The remaining part of the population was represented of small CD5+ CD21− (8%) or CD5− CD21+ (1%) lymphocytes and elements referable to neutrophils (7%). In peripheral blood the large CD5− CD21− CD11b− events represented <0.1% of leukocytes. This immunophenotypic features were suggestive of B-cell origin of neoplasia without infiltration of peripheral blood. The immunophenotype results allowed to exclude the histiocytic origin of neoplasia and to consider cutaneous B-cell lymphoma vs. CP. 

## 6. Immunohistochemistry 

Formalin-fixed, paraffin-embedded (FFPE) samples were cut into 5-μm sections, mounted on poly-l-lysine coated slides, dewaxed, and rehydrated. Immunolabeling was obtained with standard protocols on serial sections, with an antibody panel for round cell tumors including anti-CD79a, MUM1, CD20, PAX5, CD3, IBA1 antibodies, as summarized in Table 2. The immunolabeling was revealed with 3-amino-9-ethilcarbazole (Abcam); Mayer’s hematoxylin was applied as a counterstain. Negative controls were run by omitting the primary antibody incubating sections with TBS. 

**Figure 4:**
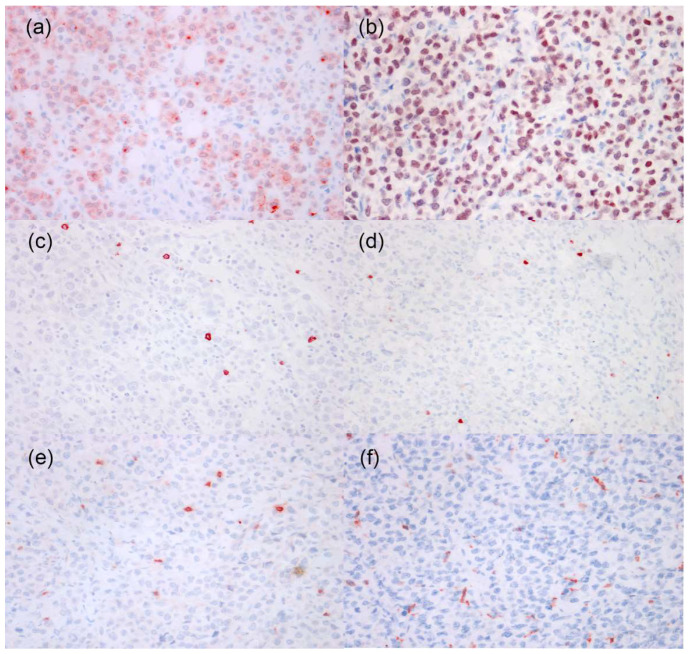
Immunohistochemistry of tumor cells in a case of in a case of canine cutaneous non epitheliotropic B-cell lymphoma (CNEBL) vs. multiple cutaneous plasmacytosis (CP). (**a**) The neoplastic cells have moderate to strong cytoplasmic expression of CD79a. CD79a (40×). (**b**) The neoplastic cells have strong nuclear expression of MUM1. IHC for MUM1 (40×). (**c**) The neoplastic cells are negative for CD20. Scattered CD20+ reactive lymphocytes are noted among neoplastic cells. CD20 (40×). (**d**) The neoplastic cells are negative for PAX5. Scattered PAX5+ reactive lymphocytes are noted among neoplastic cells. PAX5 (40×). (**e**) The neoplastic cells are negative for CD3. Scattered CD3+ reactive lymphocytes are noted among neoplastic cells. CD3 (40×). (**f**) The neoplastic cells are negative for IBA1. Scattered IBA1+ reactive histiocytes are noted among neoplastic cells. IBA1 (40×).

Immunohistochemical results (Figure 4) showed that neoplastic cells had cytoplasmic expression of CD79a, nuclear expression of MUM1, and were negative for CD20, PAX5, CD3, and Iba1. Immunoreactivity with CD3, CD20, PAX5, and Iba1 was detected in scattered round (CD3, PAX5, and CD20) or round/dendritic (Iba1) cells infiltrating among neoplastic cells, which were interpreted as reactive and inflammatory lymphocytes and histiocytes. The immunohistochemistry examination confirmed the possibility of a non-epitheliotropic B-cell lymphoma but did not rule out the possibility of a plasma cell tumor.

## 7. Chemotherapy

The chemotherapy was initiated on the basis of cytologic, histological, and phenotypical examinations suggestive of lymphoma/plasmocytoma.

Due to cardiotoxicity, doxorubicin was excluded from the chemotherapy protocol for the owner decision. Chemotherapy was initiated using a COP high dose protocol which included an 8-week induction phase (vincristine 0.75 mg/m^2^ EV once weekly, cyclophosphamide 250 mg/m^2^ PO every 21 days, prednisone 1 mg/kg/die PO for 7 days, then every other day).

Mild neutropenia occurred at the second week of induction phase and then at the fourth week, and it has been chosen to postpone the administration of drugs and then reduce its dosage (Vincristine first 0.65 and then 0.60 mg/m^2^ and Cyclophosphamide 250 mg/m^2^). Moreover, during the induction phase the patient developed an abscess lesion on the thigh that opened, with fever and neutrophilia; probably the abscess had a traumatic cause. This event forced us to stop the chemotherapy protocol until the wound was completely healed (2 weeks with local betadine medicaments and antibiotic therapy with Cefadroxil, Cefa-Cure Tabs 20 mg/kg/die OS). Because of this, the induction period is prolonged up to 10 weeks.

Contextually, painkillers (Tramadolo, Altadol 4 mg/kg/die PO) and gastric protector (Pantoprazole, Pantorc 20 mg 1 tablets/die) were administered to prevent chemotherapy-induced nausea, antiemetic drugs (Maropitant, Cerenia 2 mg/kg/die) administered the day before and the day of chemotherapy, and for 2 days thereafter. 

During the induction protocol, the patient underwent routine blood tests (complete blood count every week, hepato-renal profile every 2–3 weeks) and echocardiography and electrocardiogram were performed after 1 month from the beginning of the therapy. 

After the first vincristine administration there was an initial improvement of clinical conditions, and, as the protocol continued, the lesions were gradually not palpable and then slowly completely disappeared. 

## 8. Follow-Up 

After completing the induction protocol, the patient had regressed, and no longer presented lameness or pain of the hind limbs, the cloak was shiny, and gained 5 kg in weight. For this reason, we decided to not institute the maintenance protocol and carry on with a close follow-up every 2–3 week with clinical examination, blood test, and abdominal ultrasound.

The patient had a disease-free interval (DFI) of about 120 days; and after he had a relapse with the appearance of disseminated skin nodules with the same cytological, histological, and phenotypical features found before, in the absence of systemic signs. Due to owner financial limitations, we decided to manage relapse using a maintenance COP protocol (cyclophosphamide 250 mg/m^2^ po every 21 days; vincristine 0.75 mg/m^2^ IV every other week for 4 months, then every 21 days; prednisone 1 mg/kg/day for 7 days, then every other day until relapse or the appearance of side effects) [22].

Actually, the animal is in good clinical condition and he reached a second complete remission of 100 days (1 year from the initial diagnosis).

## 9. Discussion

We describe a rare case of CNEBL with plasmablastic differentiation vs. CP in a 12-year-old male Beagle through clinical, clinicopathological, cytological, histological, and immunophenotypical features, considering the scarce bibliographic data on these diseases.

The type of cutaneous nodules led us to include round cell tumors (lymphoma, plasma cell neoplasia and mast cell tumor other than histiocytic and melanocytic tumors) in the differential diagnosis. Cytology and histology confirmed the suspicion of round cell neoplasia and allowed to restrict the diagnosis to cutaneous non epitheliotropic lymphoma and plasma cell neoplasia. Interestingly, cytology allowed us to identify multilobate cells with clover-leaf shaped nuclei, rarely described before, that led us to consider a plasmablastic differentiation of cells. Based on two recent case reports [19,23], a suspect B-cell lymphoma with plasmablastic differentiation vs. a CP was emitted. Unfortunately, the evidence of these cells seems to be not suggestive of one or the other disease. Lee et al. [19] describe similar cells as “flower cells” in a dog diagnosed with CP, whereas Fritz et al. [23] found them in a cat with a final diagnosis of cutaneous B-cell lymphoma.

The clinical presentation of our case was similar to that reported in two other dogs diagnosed with cutaneous and subcutaneous non-epitheliotropic B-cell lymphoma [12,15]. However, in none of these cases, the markers indicative of plasmablastic differentiation MUM1, IgG, IgM and IgE were tested, nor serum protein electrophoresis was performed as was in our case. Therefore, it is possible that the diagnosis of the cases described could not be certain and, in our opinion, CP should have been included in the differential diagnosis. 

In our case, with the aim to try to make a correct and definitive diagnosis, the immunophenotype has been studied, both by flow cytometry and immunohistochemistry, by using a large panel of monoclonal antibodies never used in veterinary medicine in similar cases. The positivity to the CD79 marker, associated with the negativity to all T markers tested, indicated B-cell line neoplasia. However, the positivity of markers indicative of plasmablastic differentiation such as IgG, IgM, and IgE immunoglobulins, detected by flow cytometry, and multiple myeloma oncogene-1 (MUM1), identified by immunohistochemistry, confirmed to include CNEBL with plasmablastic differentiation vs. CP in the differential diagnosis. IgG, IgM, IgE are antigenic proteins typical both of B cells and in plasma cells [24]. On the other hand, the MUM1 is a protein that encodes a transcriptional factor thought to play a key role involved specifically in the differentiation of B lymphocytes to plasma cells [25,26]. Even if MUM1 has been detected predominantly in subjects with plasmacytoma/plasmacytosis, it is not pathognomonic of this pathology [25,27], since it has been rarely expressed also in subjects with B-cell lymphomas [25,27]. Taken together the CD79a, IgG, IgM, IgE, and MUM1 markers, may be expressed both by B-cells with plasmablastic differentiation and by plasma cells. 

Then, although flow cytometry and immunohistochemistry gave further hints to exclude the T phenotype and to confirm the B phenotype with plasmablastic differentiation of this neoplasia, they did not allow to make a final diagnosis.

Based on what above described, we reached the diagnosis of CNEBL with plasmablastic differentiation vs. CP and unfortunately, none of them could be excluded. Since related literature is very limited, we aimed to describe this case to increase information on this topic.

To the author’s knowledge, there are no previous reports of CNEBL with cytological and immunophenotypic features of plasmablastic differentiation in dogs. Conversely, in veterinary literature are only rarely reported cases of multicentric B-cell lymphoma with plasmablastic differentiation [28,29,30,31,32,33]. As in our dog, in all these cases, the diagnosis was based on cytologic features and immunophenotype results showing the positivity for markers of plasma cell differentiation (MUM 1, IgG, IgM, IgE). 

In our case, we excluded a form of multicentric lymphoma due to the absence of involvement of lymph node, splenic or hepatic, and/or blood infiltration. Nevertheless, given the impossibility to obtain a diagnosis of certainty, the patient is still under control in order to assess whether the disease evolves into a multicentric form.

**Figure 5:**
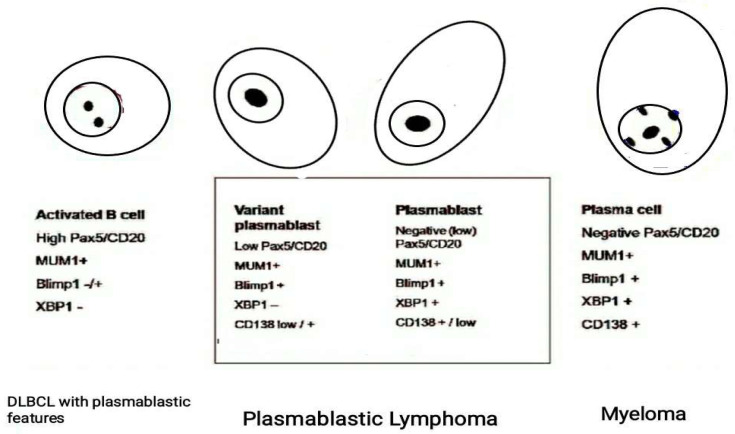
Use of a specific panel of monoclonal antibodies including PAX5/CD20, PRDM1/BLIMP1 and XBP1s useful to identify a plasmablastic immunophenotype in human B-cell Lymphomas. The varying extent of their expression defines lesions that ranges from large B-cell Lymphoma to plasmablastic lymphoma based on the degree of differentiation of B cells. [Modified from Montes-Moreno et al., 2010].

Interestingly, in human medicine, plasmablastic differentiation can be found in a variety of large B-cell lymphomas with heterogeneous clinical, histological, and genetic features, posing diagnostic challenges [34,35]. In humans, these forms are characterized by the expression of antigenic markers associated with plasmablastic differentiation (CD38, CD79a, CD138, MUM1, Blimp1, and XBP1), and a decreased expression of B-cell antigens (CD20 and PAX5) (Figure 5) [34,36]. Unfortunately, all these markers are not available in the veterinary field. 

Considering those presenting cutaneous involvement, in humans, plasmablastic lymphoma and primary cutaneous marginal zone lymphoma (pcMZL) are the most common subtypes. Plasmablastic lymphoma has systemic involvement with morphologic and immunophenotypic features overlapping with aggressive large B-cell lymphomas and multiple myeloma [34,37,38]. On the other hand, pcMZL rarely exhibits extra cutaneous spread and it is an indolent form of cutaneous B lymphoma with an excellent prognosis; with a 5-year overall survival of approximately 97% [39,40]. No standardized treatment protocol exists for pcMZL. Intralesional steroids, radiotherapy, surgery or rituximab alone or combined with chemotherapy are applied as first-line treatment [39,41]. In our case, the clinicopathological features, the response to therapy, the clinical remission and the long-time relapse could lead us to suppose a form of cutaneous non epitheliotropic B-cell lymphoma resembling the PcMZL human counterpart, even if it is not possible to state it with certainty. 

To our knowledge, there are very few studies regarding CP in dogs and they do not describe clear and exhaustive diagnostic features typical of this neoplastic form. The clinical presentation, other than the cytological and immunohistochemical characteristics, of our case are very similar to those reported in a 12-year-old female Shih-tzu dog diagnosed with CP [19]. Even if unlike in our case, the cutaneous nodules described were ulcerated and crusted, cytological presentation overlapped, with the presence of round cells with multilobate clover leaf shaped nuclei and, as in our case, the immunohistochemical evaluation indicated CD79a and MUM-1 positivity in the absence of involvement of other organs. Differently to our case that did not reveal serum mono or polyclonal peaks in the globulin zone, the case described showed hypergammaglobulinemia with a monoclonal peak in the γ region, highly suggestive, even if not pathognomonic, of plasma cell neoplasia [18,42,43,44,45,46]. Nevertheless, in our opinion, the authors should have been included in the differential diagnosis also the cutaneous B-cell Lymphoma with plasmablastic differentiation.

In a recent clinical review reporting 21 cases of canine CP with multiple and ulcerated lesions, 81% of cases were asymptomatic and indolent, and only 2 cases showed a polyclonal gammopathy. In only 8/21 cases the plasma cell origin was confirmed by immunohistochemistry through the positivity to CD79, MUM1 or λ light chain marker. The latter is one of the markers used in humane medicine to identify the plasma cell origin, but it is rarely applied in veterinary medicine since the absence of a canine specie-specific marker and the doubt cross-reactivity of the human commercial kit with the canine λ light chains [13,43]. For these reasons, unfortunately, we were not able to test this marker in our case. In addition, 30% of cases reported in this review were associated with the involvement of lymph nodes and abdominal organs [18]. In our opinion, also in this study the distinction between cutaneous B-cell lymphoma vs. CP is not clear in most of the cases included. To date, in humans, it is known that CP has a chronic and benign course, rarely fatal, with or without systemic complaints including lymphadenopathy, hepatosplenomegaly, lymphoid interstitial pneumonia, renal failure, and polyclonal hypergammaglobulinemia identified in 88–93% of patients [20,47,48,49]. The etiology of CP is poorly understood [50,51]. Poor prognostic factors include high IgG levels (>5000 mg/dL) and high plasma cell counts in the bone marrow [48,51]. In addition, the development of multicentric lymphoma and leukemia has been rarely reported [50,51,52]. A standardized treatment does not yet exist [47] and the therapy commonly used include intralesional steroid injections, oral doxycycline, chemotherapy, radiotherapy, and ultraviolet A radiation (PUVA) [50,51].

Unfortunately, the veterinary literature does not give sufficient data useful to identify distinctive clinical and pathological features that would help to distinguish certainly CP and primary cutaneous B-cell lymphoma in dogs. In human medicine, generally it is possible to obtain a precise diagnosis due to the availability of different specific diagnostic methods and markers, even if, interestingly, are also reported cases showing overlapping diagnostic features of CP and primary cutaneous marginal zone B-cell lymphoma (pcMZL) [20,50,51].

The detection and quantification of serum IgG, IgG4, IgM, the serum immunofixation electrophoresis, the immunohistochemistry(IHC) for the identification of kappa and lambda light chains of the immunoglobulins and the immunoglobulin polymerase chain reaction (PCR) for IgH gene rearrangement [43,50,51,53,54] are the techniques most used in these cases. Particularly, a recent review [53] shows that kappa and lambda light-chain restriction by IHC or in situ hybridization (ISH) are useful tools in the evaluation of cutaneous B-cell lymphomas and pcMZL neoplasms. Detection of light chains seems to support clonal atypical cutaneous lymphoid infiltrates of B-cell and/or plasma cell population. Moreover, IHC and ISH are able to identify their cellular localization allowing to distinguish B cells, that express surface light chains, and plasma cells, that have cytoplasmic light chains and lose surface light chains [53]. Unfortunately, these diagnostic markers are not available in veterinary medicine.

Regarding the response to therapy, in our case the patient had a complete remission (120 days) with a COP chemotherapy protocol, and, subsequently, he had a relapse with a second complete remission (100 days until now) using the same protocol. Survival time was more than 1 year from the initial diagnosis. Plasma cell tumors have been reported to have a better prognosis than lymphomas in dogs and they respond well to the chemotherapy protocols used in the case of B-cell lymphomas [18]. Nevertheless, a dog previously diagnosed with CP [19] and treated with COP protocol was euthanized 65 days after initial presentation due to progressively worsening of clinical condition, despite the skin lesions improved. It is worth mentioning that no other clinical case of CNEBL is reported in literature and the few cases of CP described had different treatment approaches and showed different prognosis probably due to the possible incorrect diagnosis achieved. Unfortunately, these few and confusing information present in the literature cannot aid the veterinary practitioners to achieve correct diagnosis and treatment of these infrequent diseases. 

In conclusion, even if in our case we tried to expand the immunophenotypical panel than previously reported similar cases described in dogs, we could not obtain a precise diagnosis, and we reach a final diagnosis of borderline CNEBL/CP. Based on what is reported in the scarce veterinary literature focused on these diseases and in our findings, it is common to incorrectly diagnose one or the other pathology due to the similar features and the absence of standardized specific diagnostic markers and this has crucial consequences on the diagnostic, prognostic, and therapeutic evaluation. Further studies are needed to identify specific markers able to distinguish these two entities in dogs. Some of the tests used in humans should be validated in veterinary medicine. Moreover, in similar cases a careful follow-up is fundamental to identify a possible systemic involvement and the course of pathology.

## Figures and Tables

**Table 1:** Flow cytometry antibodies used to identify cell surface antigens (CD = cluster of differentiation), with their cell type expressions, results and sources, in a case of cutaneous non epitheliotropic B-cell lymphoma (CNEBL) vs. multiple cutaneous plasmacytosis (CP) in a dog. MoAb = monoclonal antibody, Ab = antibody.

MoAb	Result	Ab Source	Cell Type Expression
CD3	−	Serotec, Oxford, UK	T cells
CD4	−	Serotec, Oxford, UK	T cells, hystiocitic
CD5	−	Serotec, Oxford, UK	T cells
CD8	−	Serotec, Oxford, UK	T cells
CD11b	+	PF Moore, UC Davis, Davis, CA, USA	granulocytes, monocytes, macrophages
CD11d	−	Santa Cruz Biotechnology, Santa Cruz, CA, USA	lymphoid and histiocytic tumor
CD14	−	VMRD, Pullman, WA, USA	Monocytes, macrophages
CD18	+	PF Moore, UC Davis, USA	Leukocyte tumors
CD21	−	Serotec, Oxford, UK	B cells
CD34	−	Santa Cruz Biotechnology, CA, USA	pluripotent haematopoietic progenitor cells
CD44	+	PF Moore, UC Davis, USA	panleucocytic
CD45	+	Serotec, Oxford, UK	panleucocytic
CD79b	+	Serotec, Oxford, UK	B lymphocytes
CD117	−	Santa Cruz Biotechnology, CA, USA	Mast cells, melanocytes, GIST tumor
IgE	+	Serotec, Oxford, UK	B cells, plasma cells

**Table 2:** Antibody table used in a case of cutaneous non epitheliotropic B-cell lymphoma (CNEBL) vs. multiple cutaneous plasmacytosis (CP) in a dog. Details of sources, concentrations, and antigen retrieval methods of antibodies used for immunochemistry in this study are described. NOAR: no antigen retrieval. Cell type expression: CD79A Mature B cells/plasma cells; MUM1 plasma cells; CD20 pre-B cells and mature B cells (<20% plasma cells); PAX5 B cells; CD3 mature T cells; IBA1 histiocytic/dendritic cells. Results: +++: the majority of neoplastic cells were positive for the investigated marker. −(+): neoplastic cells were negative but scattered immunoreactive round or dentritic cells, interpreted as inflammatory cells, were note intermixed with neoplastic cells.

Antibody	Producer	Code/Clone	Dilution	AR Method	Results
CD79a (HM75)	Santa Cruz, Oregon, USA	Sc-53208	1:200	Tris-EDTA pH 9.0	+++
MUM1	Agilent Dako, Glostrup, Denmark	M7259	1:50	Tris-EDTA pH 9.0	+++
CD20	Thermo Scientific, Fremont, CA, USA	RB-9013-P1	1:200	NOAR	−(+)
PAX5	BD Bioscience, San Jose, CA, USA	610863	1:20	Tris-EDTA pH 9.0	−(+)
CD3	Agilent Dako, Glostrup, Denmark	A0452	1:200	Tris-EDTA pH 9.0	−(+)
Iba1	Abcam, San Jose, CA, USA	Ab107159	1:250	Tris-EDTA pH 9.0	−(+)

## Data Availability

The data presented in this study are available in the manuscript.
